# Story discourse and use of mental state language between mothers and school-aged children with and without visual impairment

**DOI:** 10.1111/1460-6984.12040

**Published:** 2013-09-09

**Authors:** Valerija Tadić, Linda Pring, Naomi Dale

**Affiliations:** † MRC Centre of Epidemiology for Child Health, Centre for Paediatric Epidemiology and Biostatistics, UCL Institute of Child HealthLondon, UK; ‡ Department of Psychology, Goldsmiths, University of LondonLondon, UK; § The Wolfson Neurodisability Service, Great Ormond Street Hospital for Children NHS Foundation TrustLondon, UK

**Keywords:** visual impairment, mental state language, mother–child discourse

## Abstract

**Background:**

Lack of sight compromises insight into other people’s mental states. Little is known about the role of maternal language in assisting the development of mental state language in children with visual impairment (VI).

**Aims:**

To investigate mental state language strategies of mothers of school-aged children with VI and to compare these with mothers of comparable children with typically developing vision. To investigate whether the characteristics of mother–child discourse were associated with the child’s socio-communicative competence.

**Methods & Procedures:**

Mother–child discourse with twelve 6–12-year-old children with VI was coded during a shared book-reading narrative and compared with 14 typically sighted children matched in age and verbal ability.

**Outcomes & Results:**

Mothers of children with VI elaborated more and made significantly more references to story characters’ mental states and descriptive elaborations than mothers of sighted children. Mental state elaborations of mothers in the VI group related positively with the level produced by their children, with the association remaining after mothers’ overall verbosity and children’s developmental levels were controlled for. Frequency of maternal elaborations, including their mental state language, was related to socio-communicative competence of children with VI.

**Conclusions & Implications:**

The findings offer insights into the potential contribution of maternal verbal scaffolding to mentalistic language and social–communicative competences of children with VI.

**What this paper adds?**

Very little is known about the role of maternal language in assisting the development of children with visual impairment (VI), particularly beyond the early childhood. This study explored whether verbal input from mothers of school-aged children with VI scaffolds their children’s use of mental state language. Whilst this has been examined for typically developing children and those with other disabilities, little research has considered children who are visually impaired but otherwise cognitively and physically intact. The findings offer insight into the nature of maternal verbal scaffolding and its possible contribution to the mental and social–communicative competence of children with VI. By looking at children with VI who are cognitively unimpaired, this study also contributes to understanding the adaptive abilities of developing children and the mechanisms that can promote typical development.

## Introduction

Development of children with severe visual impairment (VI) has been associated with social–communicative and social–cognitive difficulties, including behavioural similarities with children with autism (Absoud *et al*. 2010, Brown *et al*. 1997, Fraiberg 1977, Green *et al*. 2004, Peterson *et al*. 2000, Preisler 1991, Tadić *et al*. 2010). These features, including difficulties in developing joint attention (Bigelow 2003) and perspective taking with others (Fraiberg 1977), are of considerable concern and may be evident from infancy and early years.

Research has shown the presence of an asymmetrical parent–child with VI interaction style in early childhood, where the visually impaired child’s attention cannot be directed by eye contact or gaze-following and the child’s own opportunities for following the parent’s focus of attention are seriously limited (Andersen *et al*. 1993, Preisler 1991). These early studies have also shown that mothers’ language input to their young children with VI can be highly directive, involving relatively few descriptions (e.g. on the functions and attributes of objects, events and people) (Andersen *et al*. 1993, Kekelis and Andersen 1984, Moore and McConachie 1994).

Others have reported that mothers of children with VI speak more to their children and use significantly more descriptions when interacting with the child than mothers of sighted children (Behl *et al*. 1996, Campbell 2003, Pérez-Pereira and Conti-Ramsden 2001). This suggests that parents of children with VI are able to develop alternative strategies when conversing with their children and exploit the use of language as a way to share the world with them (Pérez-Pereira and Conti-Ramsden 1999, Urwin 1978).

Despite some differing evidence concerning early mother–child interactions, the general consensus is that the language used by mothers with their children who are visually impaired differs qualitatively from the maternal language input to sighted children. However, very little is known about discourse between mothers and children with VI beyond the first 4 years of life and whether mothers play an adaptive role in supporting socio-cognitive and communicative development.

Research with young typically developing sighted children has shown that social interaction within the family unit influences children’s social understanding (Carpendale and Lewis 2004, de Rosnay and Hughes 2006, Symons 2004). Mothers’ conversational input, particularly their talk about internal mental states (i.e. talk about feelings, desires, beliefs and thoughts), has been shown to promote their child’s use of mental state language and ‘scaffolding’ the child’s theory of mind (ToM) development (de Rosnay *et al*. 2004, Meins *et al*. 2003, Ruffman *et al*. 2002, Taumoepeau and Ruffman 2006). For instance, early mental state talk by mothers has been found to predict children’s ToM outcomes at later time points in childhood, even when other potential mediators are controlled for (i.e. mothers’ overall verbosity, mothers’ educational level and frequency of other types of utterances, and children’s age, language ability, use of mental state language, and early ToM) (Meins *et al*. 2002, 2003, Ruffman *et al*. 2002, Taumoepeau and Ruffman 2006).

A similar relationship between mothers’ mental state language input and children’s success in ToM development has also been demonstrated in children with autism and children with hearing impairment (Moeller and Schick 2006, Slaughter *et al*. 2007). No studies to date have examined the impact of maternal mental state language on children with congenital visual disorders and severe VI, despite the well documented socio-cognitive difficulties in this population (Green *et al*. 2004, Peterson *et al*. 2000, Roch-Levecq 2006).

In the present study, we set out to investigate mental state language strategies of mothers of school-aged children with VI and to compare these with mothers of comparable children with typically developing vision. The advantage of examining mother–child discourse with school-aged children is that the vocabularies of older children contain a wider repertoire of mental state terms and the quality of maternal mental state talk is likely to reflect this (Taumoepeau and Ruffman 2006). To examine mother–child dialogue, the book-sharing narrative, using a standard coding system for classifying the transcribed narrative (e.g. Slaughter *et al*. 2007, Symons *et al*. 2005), was considered useful as it provides a naturalistic setting and an opportunity to elaborate and ask questions by both conversational partners, including conversation about the story characters’ thoughts and feelings and does not depend on non-verbal pictorial material. Although this method has been previously applied mainly with younger children, using the same unfamiliar book provides a standardized context for examining mother–child discourse across a broader age group of participants with and without VI.

To investigate the possible impact of maternal mentalistic language, we were interested in investigating relationships with the visually impaired child’s own use of mentalistic language and other potentially associated factors, including verbal IQ (VIQ), pragmatic language and socio-communicative competence. Studies using verbal ToM tasks with this population have shown that children with VI show an initial and continuing delay in ToM development, but VIQ can act as a moderator (Green *et al*. 2004, Peterson *et al*. 2000). Consequently, standard ToM assessment may not be sensitive to other difficulties in social understanding at school age in children with VI whose language ability is advanced. Autism research suggests that difficulties in social understanding may be more apparent in real-life situations, which may involve more subtle aspects of ToM such as appropriate use of pragmatic language and social overtures (e.g. applying conversational rules such as initiating, responding and turn-taking, maintaining meaningful conversations, and keeping track of speaker’s and listener’s mental states) (Capps *et al*. 2000, Dennis *et al*. 2001). Thus, we used parent-reported questionnaires measuring children’s everyday social interaction and communication as a standard assessment of pragmatic use of language and social competences.

Children with severe VI due to a congenital visual disorder are a rare heterogeneous population, which engenders certain methodological constraints on research studies. Study samples may include children with cerebral visual disorders of central nervous system origin, which carry a higher risk of learning difficulties (Sonksen and Dale 2002). The research has often been restricted to very small samples and individual case studies, and has generally lacked matched control groups. Thus, we felt it was important to focus on children with congenital visual disorders originating in the peripheral visual system (where there is less likelihood of central brain involvement; Dale and Sonksen 2002) and with verbal intelligence in the normal range, as well as to include an age- and ability-matched group of typically sighted children.

In summary, the study explored whether mothers of school-aged children with VI use relevant verbal input that can assist their children in understanding the mental states of others, particularly during a book-reading context where the children cannot visually access the pictorial information that is available to a typically sighted comparison group. As well as examining maternal mentalistic language input, we also explored the extent of the descriptive elaborations produced by the dyads, given some contrasting evidence concerning early descriptive language input by mothers of children with VI (Behl *et al*. 1996, Kekelis and Andersen 1984, Moore and McConachie 1994, Pérez-Pereira and Conti-Ramsden 2001). To examine the association between maternal and child mentalistic language, and in keeping with the previous sighted literature (Ruffman *et al*. 2002, Slaughter *et al*. 2007), we also made attempts to control for mothers’ overall verbosity and the child’s developmental levels (i.e. age and VIQ). Thus, the specific research questions the study addressed were:

1. Compared with the matched sighted group, do the school-aged children with VI and their mothers (a) differ in the frequency of total language output during a mother–child discourse, and (b) differ in the extent to which they refer to both internal mental states and non-mental (i.e. descriptive) elaborations during a joint book-reading narrative?
2. Is there a relationship between elaborations of mothers and those of children in the two groups? Specifically, does a relationship between children’s and mothers’ mentalistic language vary as a function of mothers’ overall verbosity and the child’s developmental level?
3. Are the characteristics of mother–child discourse associated with the visually impaired child’s socio-communicative competence?

## Method

### Participants

Twelve school-aged children with VI and 14 typically sighted children participated with their mothers.

The children with VI were identified through the database of the developmental vision clinic at Great Ormond Street Hospital, London, a national paediatric hospital, which they attended in their preschool years for management and guidance of the developmental aspects of vision loss. They all had a ‘potentially simple’ congenital disorder of the peripheral visual system (i.e. of the globe, retina or anterior optic nerve; Sonksen and Dale 2002). Children with congenital VIs of cerebral origin were excluded. Visual impairment was in the profound (i.e. light perception or worse) or severe range (basic ‘form’ vision Snellen worse than 6/36), according to functional vision measures undertaken in the clinic at the age of 5 years (Sonksen and Dale 2002). Of 20 eligible children identified for the study, twelve 6–12-year-old children without additional impairments, and for whom English was the main language spoken with their mother, were included (with informed parental consent and child assent). All but one were Braille readers at the time of the study. Their visual diagnoses were Leber’s amaurosis (*n* = 3), microphthalmia (*n* = 3), aniridia with glaucoma (*n* = 1), bilateral optic nerve hypoplasia (*n* = 2), persistent primary hyperplastic vitreous (*n* = 1), familial exudative vitreo-retinopathy (*n* = 1), and multiple opacities with sclerocornea (*n* = 1).

Informed parental consent and child assent was obtained for 17 typically sighted children recruited through local primary schools. Two children were excluded initially to facilitate group matching and one was excluded subsequently, her mother being identified as an extreme outlier in that the number of book elaborations she produced was 3.5 SDs (standard deviations) above the group mean (Howell 2012). Thus, the comparison group consisted of 14 typically sighted children most closely resembling the VI group in terms of age and VIQ.

The two groups of children were comparable in terms of their VIQ, as measured by the Wechsler Intelligence Scale for Children—III (WISC-III; Wechsler 1992) (*t*_(24)_ = 0.933, *p* = 0.360), verbal mental age (VMA) (*t*_(24)_ = 0.376, *p* = 0.710), chronological age (*t*_(24)_ = 0.051, *p* = 0.960), and gender (*χ*^2^_(1)_ = 0.004, *p* = 0.951) (table 1). The small sample size provided a limited opportunity to examine statistically the impact of other socio-demographic variables, which have been found to be related to the levels of mothers’ and children’s mental state language, such as maternal education level. Thus, we ensured that the two groups were similar with respect to these variables (table 1).

**Table 1:** Cognitive and socio-demographic characteristics of the children

	VI group	Sighted group	
	N total = 12	N total = 14	p value
Verbal IQ/VIQ			
Mean (SD)	109 (9.2)	105.5 (8.9)	n.s
Range	95–128	92–121	
Verbal Mental Age/VMA			
Mean (SD) in months	109. 3 (24.3)	106 (20.7)	n.s.
Range in years	7:02–12:10	5:11–11:10	
Chronological age			
Mean (SD) in months	101 (24.4)	100.6 (19.6)	n.s.
Range in years	6:06–12:11	6:02–11:08	
Gender			
Female	7	8	n.s.
Ethnicity			
White British	8	10	–
Black British	1	1	–
Asian	1	1	–
Mixed	2	2	–
Has siblings			
1 or more	10	12	–
		(N missing = 1)	
Birth order			
First child	5	5	–
Mother’s education level			
Further higher	6	7	–
education (e.g., college, university)	(N missing = 2)	(N missing = 1)	

Notes: n.s., Non-significant.

## Materials and procedures

### Book-sharing narrative

An illustrated children’s book *First Day Jitters*, used by Symons *et al*. (2005), was used for the mother–child book-reading session. The book depicts a character dealing with the anxiety about the first day of school and allows a discussion about mental states, as the main story theme involves a case of mistaken identity revealed at the end of the story.

All the participating dyads confirmed they were unfamiliar with the book. They were all seen at home by the first author, where they were asked to spend time reading and discussing the book together. The researcher left the room during the sessions, which were audio-recorded and took 7 min on average.

While the book reading in the VI group was conducted by the parents, the book reading was shared between the sighted children and their parents and, in a few cases, it was carried out by the children themselves. In both groups the discussion about the story events and characteristics was facilitated by the parents.

### Narrative coding

All the speech produced by the mother–child dyads was transcribed. The language that was not directly from the book was coded. First, the number of utterances relevant to the book’s content was derived for parents and children, respectively. An utterance was defined as a word or string of words identified by a pause or grammatical completeness (Symons *et al*. 2005). Second, utterances were examined for each partner and coded for the type of elaboration they contained. Although in most cases the number of utterances equalled the number of elaborations (*r* = 0.998), a distinction was made between the two because it was possible for one utterance to contain more than one elaboration.

The elaborations were classified as mentalistic and non-mentalistic. Mentalistic elaborations were coded following the criteria for mental state language by Ruffman *et al*. (2002) and Bartsch and Wellman (1995). This included references to desires (e.g. ‘*She doesn’t want to get up*.’), emotions (e.g. ‘*She seems scared*.’), modulations of assertion (e.g. ‘*I wonder why she’s hiding*.’), think and know terms (e.g. ‘*They’re thinking hard*’, excluding ‘I don’t know’ responses because of their possible use to mean ‘I can’t answer’), and other mental states (e.g. ‘*Do you remember your first day at school?*’).

Mentalistic elaborations were classified as those referring: (1) to Self (e.g. ‘*I don’t remember seeing that*.’), (2) to Partner (i.e. mother or child) (e.g. ‘*What do you think about this book?*’), (3) to Character (e.g. ‘*She thinks it’s horrible*’), and (4) Other, less specific mental state references (e.g. ‘*It’s a mind trick*’). If two different mentalistic elaborations were produced in one utterance (e.g. ‘*I think she’s scared*’), the responses were then assigned to both categories (e.g. ‘*I think*’ = self-mentalistic; and ‘*She’s scared*’ = character mentalistic).

Non-mentalistic elaborations were classified as descriptive and general, following the categories specified by Symons *et al*. (2005). Descriptive elaborations involved language referring to behavioural and physical aspects of the story and the book (e.g. ‘*the girl has short hair*’; ‘*the doggy is barking*’). General elaborations were all the other utterances that did not add descriptive value to the book-reading discourse (e.g. ‘*What’s that?*’, ‘*Let’s continue*’, etc.). On their own, general elaborations were not statistically analysed. Where utterances contained different types of elaboration (e.g. ‘*do you think her heart is beating fast or slow?*’), the elaborations were assigned to both mentalistic and descriptive elaboration categories.

Each child and mother received a score for mentalistic and descriptive elaborations, expressed as a proportion of all elaborations (e.g. proportion mentalistic = total number of mentalistic/[sum of all elaborations: mentalistic + descriptive + general]). The proportional data were considered more appropriate than frequency data as they were independent of mothers’ verbosity. The scores for each type of mentalistic reference were expressed as proportions of all mental state elaborations (e.g. proportion of self-mentalistic = number of self-mentalistic/[total number of mentalistic: character + self + partner + other]). Individually, all but other mentalistic references were considered for subsequent analyses.

An independent rater, who was unaware of the children’s characteristics or the hypotheses of the study, coded approximately 50% of randomly selected transcripts from each group, resulting in high reliability correlations overall (mother: mentalistic: *r* = 0.990 and descriptive: *r* = 0.929; child: mentalistic: *r* = 0.889 and descriptive: *r* = 0.821).

### Measures of verbal ability and social communication

The Verbal Scale from the WISC-III (Wechsler 1992) was used to assess verbal ability. Each child’s VIQ and VMA were derived from five subtests that do not require presentation of visual stimuli, and were thus suitable for use with children with VI: Information, Similarities, Vocabulary, Comprehension and Digit Span.

Children’s socio-communicative competence was assessed using the Children’s Communication Checklist—2 (CCC-2) (Bishop 2003) and the Social Communication Questionnaire (SCQ) (Rutter *et al*. 2003). These can be used with children with VI, although they have not been normed on this population. CCC-2 is a parent report-based questionnaire used to assess everyday language, communicative and socio-interactive skills across ten different subscales. Whilst not diagnostic, it can be used in screening for a potential communication disorder (e.g. autism spectrum disorder—ASD and specific language impairment—SLI). For the purposes of this study, two CCC-2 indices were used. First, to assess the use of language for social purpose, pragmatic language composite—CCC-2 PRAG—based on the sum of four CCC-2 scales assessing Context, Stereotyped Language, Non-Verbal Communication and Appropriate Initiation was derived. Second, to obtain a measure of social interaction skills, a social interaction composite—CCC-2 SOC—based on the sum of two CCC-2 scales assessing Social Relationships and Interests was derived. The higher the CCC-2 PRAG and the CCC-2 SOC composite scores, the higher the child’s competence in pragmatic language use and social interaction, respectively.

The SCQ is a parent-completed questionnaire used to screen for socio-communicative behaviours associated with ASD and which map onto the three core diagnostic domains: Reciprocal Social Interaction, Communication and the Restricted, Repetitive and Stereotyped Behaviours. The raw total SCQ score was used as a measure of socio-communicative competence (i.e. the higher the score, the lower the socio-communicative outcome).

### Ethical approval

The study was approved by the research ethics committee for the UCL Institute of Child Health and Great Ormond Street Hospital and the research ethics committee for Goldsmiths, University of London, UK.

## Results

### Data screening

Screening of the child discourse data showed one outlier in the VI group on mental states elaborations only. This child produced only one utterance, which contained a single mentalistic elaboration, resulting in the maximum mentalistic proportion score (the results we report below remain the same with this child being removed from the analyses). The maternal discourse data were normally distributed.

### Descriptive analyses

Non-mentalistic elaborations made up the largest proportion of the dyad’s language in both groups (table 2). Approximately one-third of all elaborations spoken by the mothers in both groups were those referring to mental states, compared with approximately 13% of elaborations spoken by the children. At least 40% of all mentalistic elaborations produced by mothers in both groups were those referring to their child’s mental state (Partner).

**Table 2:** The mean raw and proportion scores – for all, mentalistic (all mentalistic, and mentalistic references to self, to child and to character) and descriptive elaborations – for children and mothers in each group

Elaborations		VI	Sighted	p
Mean(SD)		group	group	value
**Mother**				
All elaborations	Raw	75.3 (48.4)	30.1 (19.5)	**
	Range	13–159	1–68	
Mentalistic	Raw	18.7 (13.11)	10.5 (7)	
	Range	2–47	0–27	
	Proportion	.27 (.11)	.34 (.13)	n. s.
To Self	Raw	4.1 (4.1)	1.9 (1.7)^a^	
	Range	0–12	0–5	
	Proportion	.19 (.11)	.16 (.12)	n. s.
To Partner	Raw	6.6 (5.9)	5.4 (3.4)	
	Range	0–19	0–13	
	Proportion	.40 (.31)	.56 (.18)	n. s.
To Character	Raw	5.5 (4.6)	1.6 (1.6)	
	Range	0–14	0–6	
	Proportion	.27 (.20)	.14 (.10)	*
Descriptive	Raw	39 (26.1)	9.1 (6.7)	
	Range	1–76	0–22	
	Proportion	.49 (.20)	.27 (.16)	**
**Child**				
All elaborations	Raw	24.8 (21.8)	14.8 (11.2)	n.s
	Range	1–56	3–38	
Mentalistic^b^	Raw	3 (3.7)	2.2 (2.9)	
	Range	0–11	0–10	
	Proportion	.15 (.28)	.13 (.13)	n. s.
Descriptive	Raw	6.1 (6.9)	4.1 (3.2)	
	Range	0–19	1–11	
	Proportion	.17 (.15)	.32 (.17)	*

Notes: n.s., Not significant; *significant at *p* ≤ .05; **significant at *p* ≤ .01.

a *N* missing = 1; One mother in the sighted group did not produce any mentalistic elaborations.

b Child data were limited with respect to the different types of mentalistic elaborations and were not included in the table.

Children generally elaborated less on the book content than their mothers, resulting in fewer data points overall. The proportion scores of mentalistic language referring to Self, to Partner (i.e. mother) and to Character could only be calculated for eight children in the VI group and for 11 children in the sighted group, as some children did not produce any mentalistic language. Thus, calculating the proportion scores for different types of mental state references was not considered meaningful for the children.

### VI versus Sighted group comparisons (research question 1)

Corrected statistics were used where variances differed significantly between the groups. Corrections for multiple comparisons were not applied because of a risk that, due to lack of statistical power, a true effect would potentially be disregarded. Cohen’s estimates of effect size ‘*d*’ have been reported for the significant results where *p* > 0.01 (Cohen 1994).

As shown in table 2, mothers of children with VI produced significantly more elaborations overall than the mothers of sighted children (*t*_(14.1)_ = 3.035, *p* = 0.009). However, there was no significant between-group difference in the overall number of child elaborations (*t*_(15.8)_ = 1.427, *p* = 0.173, *d* = 0.57).

There was no significant between-group difference in the proportions of mentalistic language spoken by mothers (*t*_(24)_ = –1.549, *p* = 0.134, *d* = –0.56) or children (*t*_(24)_ = 0.284, *p* = 0.779, *d* = 0.09). However, the proportion of references to the mental states of the story characters (Character) was significantly higher in the mothers of children with VI than the mothers of sighted children (*t*_(14.6)_ = 2.241, *p* = 0.041, *d* = 0.81). In contrast, the two groups did not differ in terms of the proportions of mothers’ child-referred mentalistic language: Partner (*t*_(17.4)_ = –1.537, *p* = 0.142, *d* = –0.62) or reference to their own mental states: Self (*t*_(23)_ = 0.649, *p* = 0.523, *d* = 0.25).

The language of mothers of children with VI contained significantly more descriptive elaborations about the book than the language of mothers of sighted children (*t*_(24)_ = 3.079, *p* = 0.005). In contrast, the sighted children’s language contained significantly more descriptive elaborations than the language of children with VI (*t*_(24)_ = –2.344, *p* = 0.028, *d* = –0.90).

### Association between mothers’ and children’s elaborations (research question 2)

Due to the limitations of child proportional data, we reverted to the raw scores for correlational analyses and used non-parametric Spearman’s rho (*r*_s_) coefficients (table 3).

**Table 3:** Spearman’s correlation coefficients (*r_s_* with *p* values in parentheses) for the relationship between mother-child discourse components and the children’s developmental levels (age and VIQ) in the VI and Sighted groups

	VI group				Sighted group			
	Child		Mother		Child		Mother	
	Mentalistic elaborations	All elaborations	Mentalistic elaborations	All elaborations	Mentalistic elaborations	All elaborations	Mentalistic elaborations	All elaborations
*Child – mentalistic*		.789**	.862**	.772**		.592*	.416	.349
*elaborations*		**(.002)**	**(.000)**	**(.003)**		**(.026)**	(.139)	(.221)
*Child – all elaborations*			.832**	.796**			.684**	.696**
			**(.001)**	**(.002)**			**(.007)**	**(.006)**
*Child age in months*	−.588*	−.812**	−.666*	−.571*	.000	.397	.315	.224
	**(.044)**	**(.001)**	**(.018)**	**(.053)**	(1.000)	(.160)	(.272)	(.431)
*WISC – III VIQ*	.612*	.262	.585*	.459	−.030	.008	.383	.387
	**(.035)**	(.411)	**(.046)**	(.134)	(.920)	(.979)	(.177)	(.171)

Note: *significant at *p* ≤ .05; ^**^significant at *p* ≤ .01.

#### VI group

As shown in table 3, the total number of elaborations produced by mothers and children in the VI group was significantly positively correlated. The frequency of mentalistic language spoken by mothers and their children was also highly correlated (table 3) and remained significant whether partialling out the mother’s verbosity (*r_s_* = 0.606, *p* = 0.046), the child’s VIQ (*r_s_* = 0.786, *p* = 0.004), or age (*r_s_* = 0.787, *p* = 0.004). The extent to which children and mothers elaborated overall (including mentalistic language) was significantly negatively correlated with the children’s chronological age.

A qualitative data example of language exchange between a 7-year-old girl with profound VI and her mother is presented below to support these results and illustrate how maternal language scaffolding for children with VI may take place (the text highlighted in bold is directly from the book):

**‘They walked to the car. Sarah’s hands were cold and clammy’ …**

Mother = : Why do you think that could be?

 Child = : I don’t know.

Mother = : Well, what makes your hands go cold and clammy? Can you think?

 Child = : When you’re sick&

Mother = : When you’re sick, yeah. What else?

 Child = : I’ve no idea.

Mother = : No idea? Do you ever get cold and clammy hands when you feel a bit nervous?

 Child = : Yeah … I felt well nervous … when I went to that music thing …

#### Sighted group

The primary purpose of table 3 is to illustrate the significant correlations for the VI group. However, there was also a significant positive correlation between the total number of elaborations produced by mothers and their children in the sighted group (table 3). Unlike the VI group, the correlation between mothers’ and children’s mentalistic language failed to reach significance (whether or not mothers’ verbosity, child’s age or VIQ was partialled out, *p*s > 0.112). However, further analyses suggested that these correlations did not differ significantly across the VI and sighted groups.^1^

Unlike the VI group, children’s age was positively, albeit non-significantly, associated with their total elaborations and their mothers’ total and mentalistic elaborations. Further analyses (using Fisher’s *Z* transformation) suggested that these correlations were of significantly different strength to the VI group (child all elaborations, *Z* = 3.46, *p* < 0.001; mother all elaborations, *Z* = 1.95, *p* ≤ 0.05; mother mental elaborations, *Z* = 2.51, *p* < 0.01).

### Association of mother and child discourse characteristics with the visually impaired child’s socio-communicative competence (research question 3)

Tadić *et al*. (2010) previously reported significantly lower outcomes on CCC-2 and SCQ in the same group of children with VI, in comparison with a larger group of sighted children, so these results are not repeated here. However, the present analysis (table 4,^2^) revealed a significant positive correlation between the maternal language input (i.e. both total and mentalistic elaborations) and children’s pragmatic language competence, as measured by CCC-2 PRAG. The number of total elaborations spoken by mothers and children correlated significantly with the children’s social competence, as measured by CCC-2 SOC.

**Table 4:** Spearman’s correlation coefficients (*r_s_* with *p* values in parentheses) for the relationship between mother-child discourse components and the children’s socio-communicative outcomes in the VI group

	VI group			
	Child		Mother	
	Mentalistic elaborations	All elaborations	Mentalistic elaborations	All elaborations
*CCC – 2*	.575	.447	.633*	.629*
*PRAG*	(.065)	(.168)	**(.036)**	**(.038)**
*CCC – 2*	.507	.755**	.572	.687*
*SOC*	(.111)	**(.007)**	(.066)	**(.020)**
*SCQ*	−.145	−.171	−.137	−.158
	(.654)	(.594)	(.670)	(.623)

Note: *significant at *p* ≤ .05; ^**^significant at *p* ≤ .01.

The total number of elaborations by mothers and children did not correlate with the child’s SCQ scores.

## Discussion

This study presents the first investigation of mothers’ verbal input to their school-aged children with severe VI during a joint book-reading context, with particular focus on the mothers’ use of mental state language. The findings showed that the maternal language input to children with VI was qualitatively different from maternal language input to the matched group of typically sighted children. Mothers of children with VI elaborated more overall and these elaborations consisted of significantly more descriptive information than the elaborations provided by mothers of sighted children. Whilst mothers of children with VI provided a similar quantity of mental state talk as mothers of sighted children, their mental state language consisted of significantly more references to the mental states of the story characters than the language of mothers of sighted children.

Approximately one-third of all elaborations produced by mothers in both groups were about mental states, showing that mentalistic language is a prominent feature of language in this age range, at least in the context of joint book-reading behaviours. Symons *et al*. (2005) reported a similar proportion (28%) of mentalistic language within the overall discourse produced by mothers during joint book-reading with their 5–7-year-old children (using the same storybook method as here). The findings suggest that this aspect of maternal language input may be an adaptive mechanism that is unaffected by their child’s sensory impairment.

At least 40% of all maternal mentalistic elaborations in both groups referred to the child’s mental state, implying that mothers generally may be sensitive towards their child’s subjective beliefs, desires and emotions (Meins *et al*. 2003); but the mothers of children with VI showed a greater tendency to refer to the story characters’ mental states than the mothers of sighted children. This suggests that these mothers may be using a compensatory strategy of tailoring their verbal input to assist their child with VI to comprehend better the invisible social world (e.g. what other people are feeling or thinking), which typically sighted children access spontaneously through vision (e.g. by observing facial expressions in the storybook pictures).

This finding may be of particular significance given the well-documented vulnerabilities in ToM development of children with VI (Green *et al*. 2004, Peterson *et al*. 2000), although we did not directly investigate the children’s ToM ability in this study. It is possible that maternal descriptions of and references to other people’s mental states may provide scaffolding on which children with VI explicitly build their mentalistic vocabulary and understanding of others. The qualitative example of a mother–child dialogue in the Results section illustrates how such scaffolding may take place. Here, the mother gradually prompts the child to relate the character’s physiological state (i.e. cold and clammy hands) with the child’s own experiences of that state and an associated mental state (i.e. feeling nervous), which culminates in the child placing her understanding of this mental state into the context of her own experiences. This type of discourse and verbal interaction is likely to occur also for typically sighted children. However, it may be instrumental in a visually impaired child’s understanding of why other people feel and behave a certain way. Thus, the findings have suggestive implications for how children with VI may develop social understanding. They also offer insight into the adaptive abilities of all developing children and the possible mechanisms that can promote typical development.

Further important insight into the possible scaffolding role of maternal language input comes from the greater number of overall elaborations, including descriptions of people, objects and events in the stories, provided by mothers of children with VI. These findings reinforce the notion that these mothers adopt alternative strategies to bring external events closer to the experiences of their child (Pérez-Pereira and Conti-Ramsden 1999, Urwin 1978). They may be particularly meaningful given some evidence of impoverished parental language input, including descriptive language, to children with VI in the early years (Kekelis and Andersen 1984, Moore and McConachie 1994).

Although children used mentalistic language much less than mothers, an important finding is that mothers’ level of mentalistic language was positively associated with children’s level of mentalistic language in the VI group. The reciprocal relationship between mother and child’s mental state language remained even after accounting for mothers’ verbosity and the child’s developmental level. Although not all correlations were significant for the sighted group, the association between sighted children’s language output and their mothers mentalistic input is in keeping with the existing sighted literature. While we cannot infer causal direction in this study, this raises the possibility that maternal language in the sighted may provide a direct facilitatory mechanism for mental state understanding. Similarly, the finding of the highly significant correlations for the VI group highlight the possible facilitatory role of maternal mental state language also in development of children with VI, as it has been shown previously in sighted children, including those with other disabilities.

Maternal verbal input was also found to relate to wider aspects of the visually impaired child’s social and communicative competences, as measured by the questionnaire measures of children’s pragmatic language and social interaction. Elsewhere, Tadić *et al*. (2010) described difficulties with pragmatic language use and socio-communicative competence in the same group of children, compared with a larger group of sighted controls of similar age and verbal intelligence. In the present study, we found a positive relationship between mothers’ elaborations on the book content (including mental state elaborations) and the visually impaired children’s pragmatic language and social interaction competence on parent-reported questionnaires. Although we cannot infer causality between maternal language input and the visually impaired child’s competence in pragmatic language use and social interaction, the findings suggest that maternal language input may have some moderating influence on the severity of these difficulties in children with VI, thus raising an important question to be addressed in future studies.

Surprisingly, we did not find a positive association with the SCQ measure, which is a screening instrument for autism. The implication of the finding is not clear yet, but it possibly complements evidence that pragmatic language difficulties may be widespread in this population even if they do not reach threshold for clinical autism (Tadić *et al*. 2010).

For the VI group, the quantity of mother–child discourse was inversely related to chronological age, suggesting that the task was biased towards younger children with higher verbal ability. Moreover the level of available functional vision in children with a severe, but not profound, VI in this study (*n* = 7) could have contributed to some variation within the VI group. Previous research has suggested that having basic ‘form’ vision in young preschool children with severe VI has significant developmental advantages compared with children with profound VI (Dale and Sonksen 2002, Moore and McConachie 1994). Because of the small sample, we combined children with different VI levels into a single group. However, the investigation of individual children revealed that the mothers of children with different levels of VI were similar in their verbal input. Although some available functional form vision (albeit severely degraded) could have allowed some children with severe VI to detect colours and general contours of larger shapes in the book illustrations, it is likely that descriptions of the characters’ facial expressions, their intentions denoted by eye gaze and many individual details of the characters’ surroundings would have been dependent on the parental observations.

The book-sharing paradigm was successful in demonstrating variation in maternal and child language in the VI group. However, in both VI and sighted groups, the frequency of child mentalistic language was relatively low. One reason for the reduced output by children with VI is that some of them were becoming too old for the task, which is consistent with the reported negative correlations with chronological age. Furthermore, the nature of the listening task may have required all the children to be passive. The reduced verbal contribution by the child could have affected the level and nature of parental involvement. Future studies would benefit from a different context for examining the mother–child mentalistic discourse such as providing the parent with a structured set of topics (e.g. about friends) and encouraging them to facilitate a discussion with their child.

Other methodological limitations include the small sample size, due to the rarity of the population, and potential bias of a clinical database sample. Loss of power may have affected the results in that some correlations were high but statistically non-significant and we were cautious about over-interpreting such findings. As with any original exploratory research, further studies testing directional hypotheses and with larger samples will be required to ensure replicability and provide further confidence in the current findings, particularly those relating to the mental state discourse. However, despite methodological limitations, assessing the characteristics of mother–child discourse during a book narrative has highlighted the positive and supportive nature of the mothers’ language to children with VI who are school-aged verbally and cognitively proficient. Although severe VI may impose constraints upon mother–child communication and language in the early years, the findings suggest that maternal input to children with VI, once children are verbally proficient, can be potentially enriching in certain contexts. Although causal relationships cannot be inferred yet, it is possible that the mothers’ verbal involvement, including their mentalistic talk, may be a strength that can be capitalized on when considering developmental interventions and guidance for parents of young children with VI. Such interventions could include parent training programmes where aspects of mother–child conversational interaction could be encouraged explicitly (e.g. verbal emphasis on mental state expressions of other people in real-life situations that the visually impaired child regularly encounters, such as on shopping trips). Implementing and evaluating such interventions at important points in development (e.g. when milestones in mental state language and social understanding are thought to emerge) may be particularly important for children with VI. Delivered at the right developmental time for the child, these interventions may target potential socio-cognitive difficulties in children with VI and facilitate their social–communicative outcomes long term.

## Footnotes

1 Both Fisher’s *Z* transformation (to compare correlations across the groups) and permutation resampling (to generate a sampling distribution under the null hypothesis and 95% CIs) were used for the difference between correlation coefficients. Neither method suggested the differences in reported correlations across the two groups were significant.

2 The aim of the analyses presented in table 4 was to address research question 3 relating specifically to the VI group by examining the association of mother and child discourse characteristics with the visually impaired child’s socio-communicative competence. However, for clarity it is worth noting that in the sighted group there were no statistically significant correlations between mother–child language components and the children’s socio-communicative competencies on SCQ, CCC-2 PRAG and CCC-2 SOC (*ps* = 0.172–0.882).
